# Supplementary material for: Understanding the growth mechanism of graphene on Ge/Si(001) surfaces
Source: Sci Rep. 2016 Aug 17;6:31639. doi: 10.1038/srep31639 (PMC4987685; doi:10.1038/srep31639)
Supplement: Supplementary Information [file srep31639-s1.pdf]

Supplementary material for the manuscript:

Understanding the growth mechanism of graphene on Ge/Si(001) surfaces

J. Dabrowski,[1] G. Lippert,[1] J. Avila,[2] J. Baringhaus,[3] I. Colambo,[4] Yu. S. Dedkov,[1] F. Herziger,[5] G. Lupina,[1] J. Maultzsch,[5] T. Schaffus,[1] T. Schroeder,[1,6] M. Kot,[1,6] C. Tegenkamp,[3] D. Vignaud,[4] and M.-C. Asensio[2]

[1]IHP, Im Technologiepark 25, 15236 Frankfurt (Oder), Germany
[2]Synchrotron SOLEIL, Saint Aubin, BP 48, 91192 Gif-sur-Yvette, France
[3]Institut für Festkörperphysik, Leibniz Universität, Appelstr. 2, 30167 Hannover, Germany
[4]IEMN, Av. Poincaré CS 60069, 59652 Villeneuve d'Ascq Cedex, France
[5]Institut für Festkörperphysik, TU Berlin, Hardenbergstr. 36, 10623 Berlin, Germany
[6]BTU Cottbus-Senftenberg, Konrad Zuse Str. 1, 03046 Cottbus, Germany

Content:

1. Figure S1: Experimental data from Fig. 4 together with the fit performed according to manuscript of Kim *et al.* [Appl. Phys. Lett. **94**, 062107 (2009)].
2. Figure S2: Comparison of the Raman spectra of the MBE- and CVD-grown graphene layers on Ge/Si(001).
3. Detailed description of the nucleation mechanism of graphene fragments on DV of Ge(001).

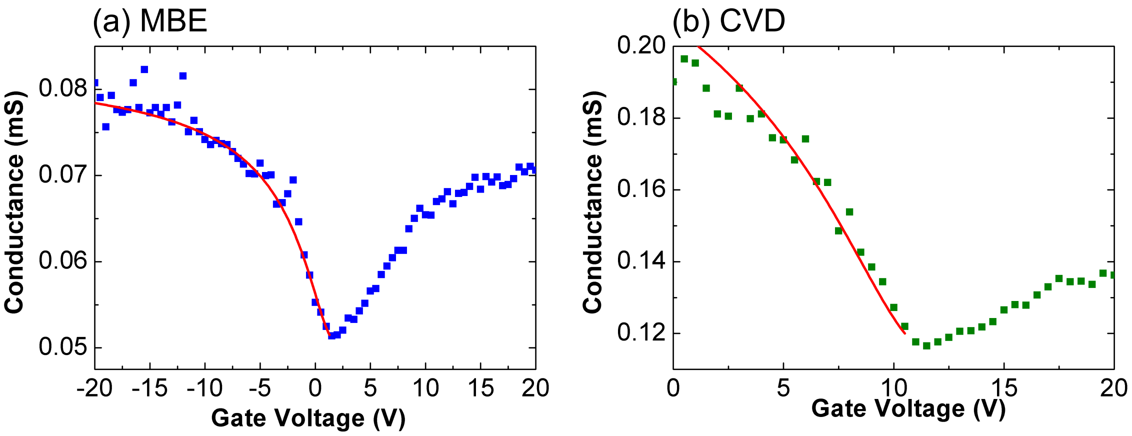

Figure S1. Experimental data from Fig. 4 together with the fit performed according to manuscript of Kim *et al.* [Appl. Phys. Lett. **94**, 062107 (2009)].

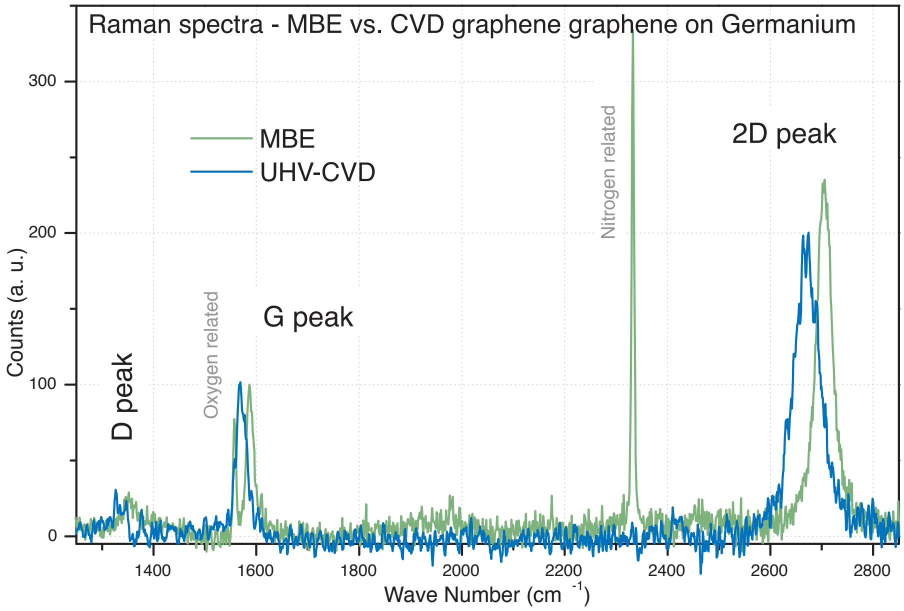

Figure S2. Comparison of the Raman spectra of the MBE- and CVD-grown graphene layers on Ge/Si(001).

Detailed description of the nucleation mechanism of graphene fragments on DV of Ge(001).

Results of our *ab initio* calculations suggest that nucleation of CVD graphene on a flat Ge(001) surface takes place predominantly on the DV defects. This conclusion follows from analysis of the reaction between C_2H_4 decomposition products.

At room temperature, C_2H_4 sticks to Ge(001). The calculated adsorption energy is however relatively low (1.2 eV), meaning that C_2H_4 desorbs easily at elevated temperatures. Moreover, the calculated diffusion barrier of C_2H_4 on Ge(001) is close to 1.2 eV: as the diffusion barrier is approached, the molecule breaks all its bonds to the substrate, so that the diffusion proceeds by subsequent desorption and adsorption steps. The conclusion is that undissociated C_2H_4 molecules have no significant direct contribution to the growth process.

The barriers for dissociative adsorption of C_2H_4 on Ge(001) equals to 0.7 eV for the hydrogen loss reaction $C_2H_4(g) \rightarrow (C_2H_3 + H)(ads)$ and 2.3 eV for the carbon dimer dissociation reaction $C_2H_4(g) \rightarrow (CH_2 + CH_2)(ads)$. In both cases, the reaction products are assumed to remain on a single Ge dimer; the separation of the products is thermally activated with the barrier close to that of the diffusion barriers if the products. The hydrogen loss reaction has much lower barrier than the carbon dimer dissociation reaction. In addition, the former reaction is exothermic (energy gain of 1.0 eV), while the latter one is endothermic (energy loss of 1.7 eV). Decomposition by hydrogen loss is therefore the dominating mechanism by which the dissociative adsorption of C_2H_4 proceeds.

The vast majority of C_2H_4 precursor molecules do not give all H atoms away to the substrate. First, transfer of more hydrogen from the molecule to the substrate is associated with energy loss and is driven by entropy. Second, most of C_2H_2 (acetylene) produced during C_2H_4 decomposition desorb before they decompose further. Thus, the major unpolymerized diffusing species expected to exist on the surface, are H and C_2H_3, with small admixture of C_2H and C_2.

These species are mobile and can react with one another. C_4H_6, which is produced when two C_2H_3 molecules meet one another, desorbs easily; the energy of gaseous C_4H_6 is only by 0.8 eV higher than that of two C_2H_3 adsorbed on the same Ge dimer. Stable polymers can be produced in other reactions, like $C_2H_3(ads) + C_2H(ads) \rightarrow C_2H_4(ads) + 1.9\,eV$. Further polymerization leads to the formation of carbon rings, by reactions like, for example, $C_4H_4(ads) + C_2H \rightarrow C_6H_4(ads) + H(ads) + 1.9\,eV$. The polymers grow in size as further elements are attached to them. Many of them remain on the surface, while the other react with the surface hydrogen and/or with C_2H_3(ads), which attack C-Ge bonds, eventually causing desorption of the affected molecules. The probability of desorption increases when the polymer becomes large enough for van der Waals forces to change its orientation from vertical to horizontal: this strains the C-Ge bonds, making them weaker.

The polymer-substrate bonds are however stronger when formed inside a dimer vacancy (Fig. 8). As an example, we consider the case of C_8H_6. This molecule is trapped at dimer vacancy (DV) defects: $C_8H_6(ads) \rightarrow C_8H_6(DV) + 1.7\,eV$. While attachment of H to C_8H_6(ads),

i. e., to the molecule sitting on a surface dimer, is energetically favorable (by 0.12 eV for the first H and by 1.32 eV for the second H), attachment of H to the trapped C_8H_6(DV) is associated with small energy loss. The probability that the molecule collects enough H atoms to desorb as C_8H_{10} is therefore much lower when the molecule is trapped as when the molecule is freely diffusing on the surface.

We conclude that graphene is produced predominantly at defect sites, such as Ge dimer vacancies.
